# Liaison psychiatry and Indian research

**DOI:** 10.4103/0019-5545.69274

**Published:** 2010-01

**Authors:** S. R. Parkar, N. S. Sawant

**Affiliations:** Department of Psychiatry, G.S. Medical College and King Edward Memorial Hospital, Parel, Mumbai-400 012, India

**Keywords:** Liaison psychiatry, GHPU, CLP

## Abstract

Liaison in Psychiatry refers to the branch of Psychiatry involving assessment and treatment in the general hospital of referred patients, like in the casualty, or patients of deliberate self farm. The Indian scene also reveals major reference from medicine, surgery, surgical super specialty and orthopedics with psychiatric disorders like anxiety, depression and / or organic brain syndromes seen in about 40 to 50 % of the medical or surgical patients. Though the Indian published data is limited, most tertiary hospitals in India carry out liaison work with various departments like Neurology, Organ transplant, Intensive Care Units and Cosmetic Surgery, so as to give comprehensive health services to patients. Liaison in Psychiatry has thus brought the emphasis on the teaching of psycho-social aspects of medicine and also increased research possibilities.

## INTRODUCTION

Mental health consultation is the need of the hour and has been emphasized on since time immemorial.[1] In the beginning, medical professionals reacted unfavorably to the admission of psychiatric patients in general hospitals. However with recognition of organicity and superimposed psychological reaction to medical illness in medical and surgical departments, psychiatry was appreciated and eventually integrated.[2] The rapid growth of general hospital psychiatric units all over the world has provided impetus to consultation-liaison work carried out by the psychiatrists. Consultation liaison psychiatry (CLP) was generally limited to the diagnostic, research and therapeutic activity in the non psychiatric departments of the general hospital. It is synonymous to liaison psychiatry (psychiatric dictionary, Oxford 1970). The mainstay CLP reports are to recommend the referrer basic logical clinical findings, appropriate treatment and follow-up programs. In addition, other objectives are educational, ethical and medico-legal. Though general hospital psychiatry was considered to come to light as a result of the lack of sufficient funds to initiate new lunatic asylums, today it is acknowledged as a major part of the public health system that takes care of mental health problems of a large population. Today, Liaison Psychiatry has acquired the status of a subspecialty within psychiatry and this has helped shift psychiatry from mental hospitals to a general hospital setting. This has also increased referrals from the non-psychiatric departments and given the psychiatrist an opportunity to directly deal with the physically ill.[3] In general, there is no specific philosophy or particular clinical context being identified in Liaison Psychiatry, at present, in India.

### Referral types

Several researchers have found a lower referral rate in the Indian counterparts as compared to the western figures. Jindal *et al*.(1980) found a poor referral rate in their study as compared to other studies conducted in India.[4] The in-patient referral rate in their study was 0.15%, as compared to 1.4%. in the study by Prabhakaran (1968) and 0.66% in the study by Parekh *et al*. (1968).[4–6] Chatterjee and Kutty (1977) reported a referral rate of 2.64% among the out-patients as compared to 0.06% by Jindal *et al*.[7,4] Most studies quote about 60% of referrals from general medicine and 14% from surgery and surgical super-specialties.[4–6]

There is also a paucity of data concerning psychiatric emergency referral in the Indian setting. Most of the available studies pertain to the routine inpatient referral. Though psychiatric services are available in almost all teaching general hospitals in India, little is known as to why the psychiatrist is called in emergency situations and what is the magnitude of the problem. Kelkar *et al*. (1982) found suicidal attempt (13%), excitement and violence (10%) and altered sensorium (9%) which constituted 32% of the total emergency referrals.[8] In the study by Gautam (1978) a vast majority (88%) of the sample of patients who presented with somatic symptoms were neurotics.[9]

### General hospital psychiatry units

General hospital psychiatry units have provided increased opportunities for interaction between psychiatrists and other medical specialists, making consultation-Liaison Psychiatry more meaningful. The establishment of General Hospital Psychiatry Units (GHPU) proved an impetus for Indian studies on psychiatric morbidity in medical-surgical inpatients.[10] Among the first units of this nature were those of R. G. Kar Medical College and Hospital, Calcutta and Grant Medical College and J.J. Group of Hospitals, Bombay, started in 1933 and 1938 respectively. By 1970, about 90 psychiatric clinics were operative in India (Directory of Mental Health Services in India, 1970). The spectrum of psychiatric case material seen in general hospital psychiatry units is much wider than seen in mental hospitals. Unlike mental hospitals, where the clinical material is predominantly psychosis, in a general hospital psychiatry unit there is a wide range of clinical problems including psychoses, neuroses, personality disorders, drug dependence and organic brain disorders.[11–13] Referral from inpatient services offers additional area for study in psychosomatic illness.[14] Malhotra S (1984), in her study, found that it was not simply the presence of abnormal behavior that prompted psychiatric consultation, but other reasons like organic illness insufficient to explain symptoms.[3] The trends worth noting were, however, a low representation of personality disorders and drug/alcohol dependence in their study with a uniformly low representation of psychosomatic disorder also. The possible explanations for this may be the focus on classical psychiatric disorders and not the personality disorders which are prevalent currently, due to various classification systems like Diagnostic and Statistical Manual of Mental Disorders (DSM) and International Classification of Diseases (ICD).

A high prevalence of psychiatric morbidity amongst general hospital OPD patients was reported in some studies in India, (36%) Krishnamurthy S *et al*. (1981)and (10.4%) by Sriram *et al*. (1987).[15,16] R.S. Murthy (1998), in his editorial, stated that the developments in the twentieth century have dramatically changed concepts of mental healthcare as a result of new knowledge and has seen a shift from mental illness to mental health.[17]

Bhogale *et al*. (2000) found that 47.57% of indoor referrals and 62.75% of outdoor referrals had unexplained physical symptoms.[18] This group also included those patients who had co-existing physical illness but symptoms were disproportionate to the physical condition. Analysis of final diagnoses in this study discovered that a large majority of the patients had neurotic, stress related, somatoform disorders (indoor 36.76% and outdoor 52.29%) followed by mood disorders (indoor 21.08% and outdoor 18.95%). The authors suggest that more interaction and dialogue between psychiatric team and referring physician is a need; their study highlighted that types of patients referred in multi specialty hospitals are vastly different and the present post-graduate training in psychiatry and psychology was inadequate in this area.[18]

### Diverse research studies

There are some diverse studies which have been reported in the Indian Journal Psychiatry. They are infrequent from those that follow diagnostic profile. They are worth noting in the Indian context. In the general hospital, in the psychiatric clinic, N. N. Wig (1968) reported cases of post vasectomy syndrome; the common pattern being that of a chronic and disabling neurasthenic hypochondriac state.[19] However, till date, these aspects have not been researched in Indian Psychiatry. There is some research documentation from army set up in IJP. A survey, by Major R. S. Mathur (1977,) of 638 soldiers hospitalized for physical illnesses or trauma in a military hospital has revealed psychiatric morbidity in 34.5% of them, manifesting mainly in states of depression (47.9%) and anxiety (40.9%).[20] Psycho-neuroses without obvious depression or anxiety formed 11.4%. The subjects who showed psychiatric morbidity with their somatic illnesses had a longer hospitalization period than the others. Positive correlation of psychiatric morbidity in physical diseases has been noticed with certain diagnostic categories, literacy level and certain states of residence; and no correlation has been seen with age, marital status, and length of service or rank of the subjects. In some cases of intra-cranial space occupying lesions, infections and cerebral seizures, who either presented as psychiatric problem or developed mental symptom, an attempt was` made to discuss the pathophysiology of psychiatric symptoms in organic brain diseases.[21] Dash and Dash (1979) found that despite advice of termination of pregnancy in certain vulnerable patients on psychiatric grounds, only 56 per cent of them accepted medical advice.[22] These patients were better educated, hailed from urban areas and belonged to higher strata of society than those who rejected such an advice. Comparison of diagnoses in the 1967 and 1977 groups showed marked differences with decrease in the epilepsy and organic brain syndromes due to the development of a neurology department.[23]

The proportionate number of schizophrenics in the clinic population has more than doubled over the 10-year period. This is possibly because of better awareness. Indian Research on liaison work is mostly with cardiology, dermatology, orthopedics, gynecology, medicine, gastroenterology and ophthalmology. Thus there is a great scope for combined service and training programs with other specialties like internal medicine, pediatrics, neurology, obstetrics and gynecology. In fact, there is hardly any clinical specialty which is not related to psychiatry or with which psychiatry cannot combine, to organize a program.

New avenues are coming up daily with the introduction of new services where psychiatric aspects are of great importance in a general hospital. Cardiac surgery, epilepsy surgery, cosmetic surgery, dialysis units, kidney transplants, intensive care units and family planning services are some of the examples in this growing field. Chandra has done extensive work in the area of women’s mental health in general and specifically the area of the interface between psychiatry and women’s reproductive and sexual health with far reaching clinical and social consequences.[24] Comparatively, a lot of work has been documented in the area of deliberate self harm and suicide in Indian set-up. One critical finding by R.K. Chadda and S. Shome (1996) is that psychiatric consultation services are not sufficiently utilized by a large number of clinicians.[25] Most of them felt the need to improve upon undergraduate medical education in psychiatry in India as well as a desire to have consultation - liaison psychiatric units in India. In an interesting study by P. Gopala Sarma (2000), on patients attending general hospital psychiatry out-patient (OP), the cost of one visit was Rs. 201. The management’s contribution to the total expenditure was 68% and patients’ 32%. Salaries accounted for the maximum - 48%. This was followed by loss of earnings -17%. Drugs accounted for less than 10%.[26]

Liaison Psychiatry has brought the emphasis on the teaching of psychosocial aspects of medicine in diverse manners like bedside interviews, interdepartmental case conferences. Research possibilities are unlimited. There are many examples of psycho geriatric clinics and memory clinics in operation in general hospital psychiatry set-ups in India and data from these set-ups will be useful in guiding these special services. Numerous studies on the psychosocial aspects of physical illness and new medical and surgical procedures, such as chronic hemodialysis, open heart surgery, organ transplantation doctor-patient relationship; stress and coping strategies; psychological antecedents of illness and many other relevant clinical problems have been carried out.[3] In all probability, an even more important need of research in the area of liaison psychiatry is to put together a “client profile” and develop tailor made services in the most advantageous way. It is acknowledged that these services are acceptable to people and there by will be able to reduce stigma related to Psychiatry. In future, however, there is a need to look at cost effective planning of these services as well as the role of socio-cultural and biological parameters in liaison psychiatry.
